# Relationship between Bilateral Landmarks of Facial Asymmetry in Skeletal Class II and Class III in Vertical Dimension: 3D Facial Scan and Cone-Beam Computed Tomography

**DOI:** 10.3390/diagnostics14060590

**Published:** 2024-03-11

**Authors:** Tanapat Jearanai, Bancha Samruajbenjakun, Pannapat Chanmanee

**Affiliations:** Orthodontic Section, Department of Preventive Dentistry, Faculty of Dentistry, Prince of Songkla University, Hat Yai, Songkhla 90110, Thailand; tanapat.j.psu@gmail.com (T.J.); samruaj@hotmail.com (B.S.)

**Keywords:** cone-beam computed tomography, diagnostic imaging, facial asymmetry, three-dimensional imaging

## Abstract

Significant facial asymmetry can lead to both functional and aesthetic issues. Often, such asymmetry originates from irregularities in the jaw structure. It is critical to recognize that asymmetries can be concealed by soft tissue, which may be overlooked. The aim of this study is to investigate the relationships between bilateral landmarks in the vertical dimension of facial asymmetry among individuals with skeletal Class II and Class III malocclusions. Fifty-two adult patients with a mean age of 24.4 ± 3.79 years with facial asymmetry who underwent orthodontic and orthognathic surgery were studied. Cone-beam computed tomography and facial scans were used to create 3D virtual head models which enhanced the accuracy in addressing facial asymmetry to ensure effective treatment planning. Each landmark was measured and digitized using the Dolphin Imaging program. The findings showed a correlation between gender and type of skeletal discrepancies with the menton deviation direction. In conclusion, the vertical discrepancy between bilateral landmarks was observable in both hard and soft tissues with more pronounced expression in soft tissues. This discrepancy was characterized by an elevation on the same side as the menton deviation, which was a feature observed in both skeletal Class II and Class III individuals.

## 1. Introduction

Many parts of the human body are characterized by bilateral symmetry, which allows them to be divided into identical mirror images on both the right and left sides. However, perfect facial bilateral symmetry is rarely seen [1,2,3] due to inherent biological factors in development and environmental influences. In the past, mild facial asymmetry was often overlooked as a normal characteristic of the face. Lately, even slight asymmetries have started to cause concerns and complaints from patients. Additionally, significant facial asymmetry can lead to both functional and aesthetic issues [4,5]. Enquist and Arak [6] highlighted a notable correlation between symmetry and attractiveness in both animals and humans. Furthermore, Choi et al. [7] categorized facial asymmetry based on its directional vector. They identified horizontal asymmetry through chin deviation while vertical asymmetry was typically marked by noticeable occlusal canting.

Facial asymmetry is often a result of deviations in the jaw that include the maxilla and mandible, which represent common dentofacial irregularities. This asymmetry is typically more pronounced in Class III patients due to pronounced chin projection. Numerous studies investigated the prevalence of left-sided menton deviation in skeletal Class III malocclusion patients who exhibited facial asymmetry [8]. In contrast, other studies reported the absence of such trends in individuals with skeletal Class III malocclusion, particularly in patients with elongated facial structures [9]. Nevertheless, it is crucial to recognize that asymmetries masked by soft tissue coverage in Class II patients might be overlooked. Overlooking these could result in incorrect diagnoses, prolonged treatment durations, and suboptimal outcomes [10].

A contributing factor to this facial skeletal asymmetry could be the various types of skeletal relationships. Kim et al. [11] indicated a potential link between the angle of the condyle and the growth patterns of the mandible. If growth in any of these areas becomes obstructed or compensated, it can lead to asymmetrical growth, and development within the craniofacial skeletal may result in a deviation of the chin from the mandibular midline [12].

Significant facial skeletal asymmetries frequently necessitate a combination of orthodontic interventions and, where applicable, maxillofacial surgery. In cases involving adults with skeletal anomalies, a dual approach involving both orthodontic and surgical methods is typically essential [13]. Evaluation of facial asymmetry is all-encompassing, incorporating a comprehensive patient history, physical examination, and biomedical imaging. These components are crucial for an accurate diagnosis, in-depth analysis of asymmetry, and formulating a tailored treatment strategy [14]. Within the scope of orthognathic surgery, the three key tissue groups, namely facial soft tissues, facial skeleton, and dentition that are collectively known as the tissue triad [15], are assessed for their contributions to facial asymmetry [5].

When evaluating facial asymmetry, it is imperative to consider any compensatory postures adopted by the patient as these may influence the accuracy of the assessment [16]. Consequently, a thorough and detailed analysis is indispensable. The assessment typically begins with an extraoral examination that often incorporates 2D photography. However, technological advancements have facilitated the development of more sophisticated methods. The employment of cone-beam computed tomography (CBCT) in conjunction with facial scanning has become increasingly common [17,18,19]. This combination enables the creation of a three-dimensional (3D) virtual model of the head [20,21] that markedly enhances the evaluation and planning phases by providing a more accurate and comprehensive view of facial asymmetry [15].

The aim of this study was to investigate the relationships between bilateral landmarks in the vertical dimension among patients who exhibited facial asymmetry with differing skeletal discrepancies, specifically those classified as skeletal Class II and Class III.

## 2. Materials and Methods

This study was approved by the Human Research Ethics Committee (HREC), Faculty of Dentistry, Prince of Songkla University (Reference number: EC6602-006). Informed consent was obtained from all subjects involved in the study. Sample size was calculated by G*Power software (version 3.1.9.7, University of Kiel, Kiel, Germany). The level of significance was set at 95% (α = 0.05), the calculated effect size was 0.80, and the power of test (1-β) was set at 80% based on our pilot study to detect the mean difference between bilateral landmarks. Twenty-six subjects per group were required.

### 2.1. Patient Inclusion and Exclusion

This study employed a cross-sectional analytical approach that included adult patients aged 18–35 years who exhibited facial asymmetry and were candidates for combined orthodontic treatment and orthognathic surgery from January 2022 to October 2023 with no history of orthodontic treatment or current orthodontic appliances. The exclusion criteria were patients with craniofacial syndromes, cleft lip and palate, history of systemic diseases or medications that would affect facial skeletal development, and patients who had undergone plastic, cosmetic, or orthognathic surgery in the head and neck area. Based on these criteria, the study enrolled 52 CBCT images and facial scanning. These patients, who were categorized into two groups (skeletal Class II and Class III) of 26 each, were identified for combined orthodontic and orthognathic surgery treatments according to their skeletal Class II and Class III dentofacial deformities. Pre-operative CBCT and facial scans were utilized to generate 3D representations of craniofacial structures to aid in accurate diagnoses and effective treatment planning.

### 2.2. Data Acquisition and Assessment

The CBCT and facial scans were captured using a CBCT scanner (Planmeca Viso^®^ G7, Planmeca Oy, Helsinki, Finland) with a resolution of 400 µm and a field of view of 30.0 × 30.0 cm, 120 kV, 8.0 mA, and 1155 mGy × cm^2^. During the scanning process, the patients were positioned standing upright in a natural head posture. They were instructed to bite in their intercuspal position and keep their lips relaxed and in a neutral position. This setup ensured the acquisition of precise and accurate 3D representations of the craniofacial structure.

**Figure 1:**
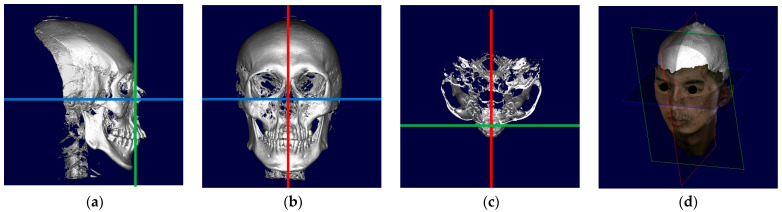
Images of the orientation settings of a virtual head: (**a**) horizontal reference line (blue line); (**b**) vertical reference line (red line); (**c**) sagittal reference line (green line); (**d**) final construction of reference planes in a 3D view.

The reference planes were modified from previous studies [22,23,24] (Figure 1). The horizontal reference line (HRL) was defined as the line connecting the right and left orbitales, and the line perpendicular to the HRL passing through the anterior nasal spine (ANS) was defined as the vertical reference line (VRL). The sagittal reference line (SRL) was defined as the line connecting the anterior nasal spine (ANS) and posterior nasal spine (PNS). After setting all reference lines, we obtained the 3D virtual head images for assessment.

**Figure 2:**
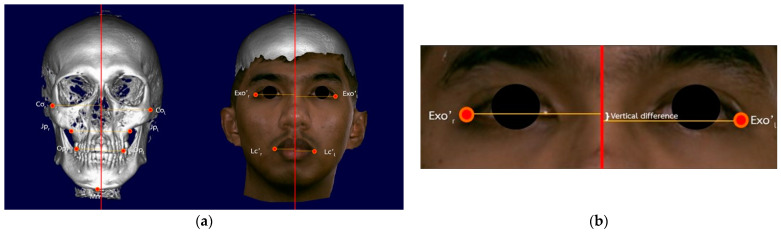
(**a**) Landmarks of soft and hard tissue as the menton (Mn), condylar head right and left (Co_r_ and Co_l_), jugal process right and left (Jp_r_ and Jp_l_), occlusal plane right and left (Op_r_ and Op_l_), exocanthion right and left (Exo’_r_ and Exo’_l_), and lip commissure right and left (Lc’_r_ and Lc’_l_) and (**b**) measurement of the difference of bilateral landmarks in the vertical direction.

The landmarks and measurements used in this study are summarized in Figure 2a. The 3D models were registered, fused, digitized, and analyzed by Dolphin Imaging program version 11.9 (Dolphin Imaging System; Canoga Park, Los Angeles, CA, USA). Lines were drawn from each landmark to the vertical reference line. The differences in the vertical dimensions were measured by the same investigator (Figure 2b).

### 2.3. Statistical Analysis

Descriptive statistics for the mean and standard deviation were calculated using the SPSS program version 26 (SPSS, IBM Corp., Armonk, NY, USA). The data were tested for normality of distribution by the Kolmogorov-Smirnov test (*p* < 0.05). The results indicated a normal distribution. Paired *t*-tests were used for inter-group comparisons to detect differences between the skeletal Class II and Class III groups. Pearson’s correlation analysis was used to assess the relationships between the menton deviation and differences in the vertical parameters. Pearson’s chi-square test, Mann–Whitney U test and Spearman’s correlation were used when the data were not normally distributed.

## 3. Results

This study included 52 patients, which was comprised of 27 females and 25 males. The mean age of the patients was 24.4 ± 3.79 years. The average measured menton deviation was 3.76 ± 0.99 mm. The findings indicated that a high percentage of the females (18/27) exhibited menton deviation to the right, and the majority of the males (15/25) showed deviation to the left. Additionally, the study found a correlation between skeletal type and the direction of menton deviation. This finding indicated that individuals with skeletal Class II were more likely to have menton deviation to the right (18/26), while those with skeletal Class III predominantly exhibited menton deviation to the left (16/26) (Table 1).

Table 2 reveals significantly higher percentages (67.3–86.5%) of the bilateral landmarks on the same side as the menton deviation in both skeletal Class II and Class III patients that included 52 individuals. The occlusal plane was most frequently observed (86.5%) to be higher on the same side as the menton deviation, which demonstrated a consistent trend across the patient population studied.

No significant differences were observed in the higher percentages of the bilateral landmarks on the same side as the menton deviation between skeletal Class II and Class III malocclusions (Table 3). This suggested a similar pattern between the side of the menton deviation and the elevated side of the bilateral landmarks in both Class II and Class III patients. Notably, in skeletal Class II, the exocanthion was more frequently elevated (88.5%) on the same side as the menton deviation, whereas in skeletal Class III patients, this pattern was predominantly observed in the occlusal plane (88.5%) (Table 3). This consistency across different skeletal classes underscores a fundamental characteristic in the manifestation of facial asymmetry.

Table 4 details the measurements of the vertical differences in the bilateral landmarks. The condylar head, jugal process, and occlusal plane constituted the hard tissue landmarks, while the exocanthion and lip commissure were classified as soft tissue landmarks. Skeletal Class II presented the highest vertical discrepancy at the exocanthion (1.20 mm) and the lowest vertical discrepancy at the jugal process (0.40 mm). On the other hand, skeletal Class III presented the highest vertical discrepancy at the condylar head (1.45 mm) and the lowest vertical discrepancy at the jugal process (1.08 mm). However, no statistically significant differences were observed in the vertical discrepancies between skeletal Class II and Class III patients, which indicated a consistent pattern of vertical asymmetry across these two classes.

From the correlation analysis, the menton deviation and various vertical difference parameters are detailed in Table 5. The menton deviation was moderately negatively correlated with the jugal process difference (r = −0.493, *p* < 0.01), whereas the menton deviation was weakly positively correlated with the occlusal plane differences (r = 0.337, *p* < 0.05). Additionally, the condylar difference was weakly positively correlated with the commissure difference (r = 0.316, *p* < 0.05).

## 4. Discussion

In analyzing patients with facial asymmetry, a significant correlation was found between menton deviation and gender. The menton deviation occurred predominantly to the right in females while males showed a higher incidence of deviation to the left. This observation aligns with previous studies [25,26], which indicated that females generally have a larger mean area on the right side of the face whereas males tend to have a larger area on the left side. This right-side dominance in females may be related to neuroanatomic development [27] that potentially explains the natural imbalance in growth between the right and left sides of the face. These findings imply that asymmetry is an inherent feature of human facial structure [28].

Kawamoto et al. [29] categorized the etiologies of an idiopathic laterally deviated mandible into two distinct groups. The first category encompasses alterations in the cranial base and glenoid fossa that result in the displacement of the mandible and condylar head. This condition leads to variable levels of the condylar head, which is a phenomenon that aligns with the findings of this current study. The second category relates to anomalies in the condyle itself that potentially lead to either underdevelopment (hypoplasia) or overdevelopment (hyperplasia) of the condyle. Consequently, when the mandible displays deviation, it contributes to lip canting and deviations in other facial landmarks. This phenomenon was observed in 2D imaging studies conducted by Lee [30] and Hwang [31], and was further substantiated through 3D imaging research conducted by Kim [32].

Numerous studies [31,33,34] have reported a notable correlation between the horizontal inclination of the lip line and canting of both the maxillary base and mandibular gonial angle. These correlations are recognized to be influenced by various factors, including the alignment of the occlusal plane, maxillary canting, discrepancies in the lengths of the two mandibular rami, and the extent of menton deviation. These findings suggest that changes in the lip line and the angular relationships of the maxillary and mandibular structures can serve as indicative markers of facial asymmetry. Furthermore, these studies observed that deviation of the mandibular chin point significantly impacts the assessment of facial asymmetry. These observations align with the results of our study, which identified significant discrepancies in all bilateral landmarks with the elevated side corresponding to the side of menton deviation.

The findings of this study emphasize that, in both asymmetrical skeletal Class II and Class III patients, menton deviations are not the sole concern. Disharmonies in other craniofacial aspects, such as the vertical differences in bilateral landmarks, were also apparent. As noted by Thiesen et al. [10] in the case of Class II patients with facial asymmetry, chin deviation exhibited a significant correlation with lower dental midline deviation, variations in jugal vertical displacements, and discrepancies in gonion lateral positions. Conversely, a study by Cho et al. [35] on skeletal Class III patients with asymmetry reported notably smaller condylar and ramus volumes on the deviated side.

This current study highlights that facial asymmetry can manifest in both the hard and soft tissues that collectively contribute to facial appearance. Therefore, a comprehensive diagnosis of facial asymmetry necessitates the evaluation of both skeletal and soft tissues in the craniofacial region. Some research findings [36,37] suggest that the soft tissue components overlaying craniofacial structures may compensate for underlying skeletal asymmetry. Furthermore, Masuoka et al. [38] reported instances of significant skeletal asymmetries when assessed through posteroanterior radiographs in patients who were clinically categorized as asymmetric or mildly asymmetric. In cases where a discrepancy arises between skeletal measurements and subjective evaluations, it is imperative to consider the influence of soft tissue structures in the context of facial asymmetry. Consequently, a thorough clinical examination in such patients should be complemented with other valuable diagnostic tools, including dental models, photographs, radiographs, tomography, and potential bone scintigraphy in specific cases, to precisely locate and measure the structures contributing to the asymmetry [16].

The current literature [39,40,41] clearly indicates a consensus among clinicians regarding the importance of 3D imaging, image fusion, and the use of 3D virtual head models in the assessment and planning stages of treatment. This current study introduced an approach to explore the association between hard and soft tissues that revealed a significant correlation between the direction of menton deviation and the corresponding elevation of bilateral landmarks. This correlation is not only apparent in hard tissue but is even more distinct in soft tissue landmarks. Thus, in devising treatment plans for facial asymmetry, it is essential to give equal consideration to both soft and hard tissues. Precise evaluation is vital for an accurate diagnosis and effective treatment planning. Reyneke et al. [42] advocated for a system that evaluates the positions of three anatomical areas (i.e., the maxilla, mandibular body, and mandibular symphysis) in relation to the facial midline and the presence of occlusal canting. Employing this system aids in determining the most appropriate orthodontic and surgical methods for each specific asymmetry type.

Investigations of facial asymmetry arising from discrepancies between hard and soft tissues, including their respective alignments, are influenced by the canting or unevenness of bilateral landmarks and menton deviation [31,34,43]. Setting the midsagittal plane plays an important role in facial asymmetry research because asymmetry and its extent are determined in relation to this plane [44,45]. Additionally, employment of horizontal reference lines is of significance. In 2D photographic analysis, the exocanthion is frequently utilized as a horizontal reference, but this may not accurately reflect the patient’s natural head orientation. Dependence on this reference for measurements or treatment planning could result in a misdiagnosis or a flawed planning strategy. Therefore, the most essential element is the accurate orientation of the 3D virtual head model to closely mirror the patient’s natural head posture.

In this study, we faced limitations in terms of geographic and ethnic diversity because it primarily focused on a Southeast Asian population. Future studies should evaluate the extrapolation of our findings to other ethnic groups and geographic areas.

## 5. Conclusions

The vertical differences in bilateral landmarks was evident in both hard and soft tissues with a more pronounced expression in soft tissue. The elevated side typically corresponded to the side of the menton deviation. Skeletal Class II patients tended to have chin deviation more to the right, while those with skeletal Class III deviated predominantly to the left.

The clinical implication of this study highlights the benefit of employing 3D virtual head models, which facilitate enhanced assessment and planning for orthodontic treatments combined with orthognathic surgery to improve communication and understanding between orthodontists and surgeons. This approach allows for effective treatment planning for patients with facial asymmetry in skeletal Class II and III discrepancies, aspects that were previously prone to being overlooked.

## Figures and Tables

**Table 1:** General characteristics of the patients including skeletal type and the menton deviation direction.

	Total, *n* = 52	Skeletal Class II, *n* = 26	Skeletal Class III, *n* = 26
Age (year, mean ± SD)	24.40 ± 3.79	24.43 ± 4.30	24.37 ± 3.29
Menton deviation (mm, mean ± SD)	3.76 ± 0.99	3.95 ± 1.09	3.58 ± 0.87
Side of menton deviation			
Right	28	18	10
Left	24	8	16
Gender			
Female	27	16	11
Menton deviation to the right	18	13	5
Menton deviation to the left	9	3	6
Male	25	10	15
Menton deviation to the right	10	5	5
Menton deviation to the left	15	5	10

SD: standard deviation.

**Table 2:** Percentages of bilateral landmarks on the same side as the menton deviation.

Bilateral Landmarks	Same Side	Other Side	*p*-Value
Condylar head	73.1%	26.9%	0.001 *
Jugal process	67.3%	32.7%	0.008 *
Occlusal plane	86.5%	13.5%	<0.001 *
Exocanthion	80.8%	19.2%	<0.001 *
Lip commissure	75.0%	25.0%	<0.001 *

* Statistically significant difference by Pearson chi-square.

**Table 3:** Percentages of the bilateral landmarks on the same side as the menton deviation in skeletal Class II and Class III.

Bilateral Landmarks	Skeletal Class II, *n* = 26	Skeletal Class III, *n* = 26	*p*-Value
Condylar head	76.9%	69.2%	0.092
Jugal process	69.2%	65.4%	0.777
Occlusal plane	84.6%	88.5%	0.165
Exocanthion	88.5%	73.1%	0.262
Lip commissure	76.9%	73.1%	0.402

**Table 4:** Measurements of vertical differences in bilateral landmarks.

Vertical Difference in Bilateral Landmark (Millimeter)	Skeletal Class II	Skeletal Class III	*p*-Value between Class II and III
Hard tissue landmarks			
Condylar head (Co_r_-Co_l_)	0.91 ± 2.00	1.45 ± 1.57	0.510
Jugal process (Jp_r_-Jp_l_)	0.40 ± 2.98	1.08 ± 1.96	0.707
Occlusal plane (Op_r_-Op_l_)	0.98 ± 1.08	1.34 ± 0.69	0.472
Soft tissue landmarks			
Exocanthion (Exo’_r_-Exo’_l_)	1.20 ± 1.58	1.34 ± 1.62	0.706
Lip commissure (Lc’_r_-Lc’_l_)	0.94 ± 1.33	1.32 ± 1.08	0.487

**Table 5:** Correlation coefficients between the menton deviation and vertical parameters.

	Menton Deviation	Condylar Difference	Jugal process Difference	Occlusal Difference	Exocanthion Difference	Commissure Difference
Menton deviation	1					
Condylar difference	−0.031	1				
Jugal process difference	−0.493 **	0.223	1			
Occlusal difference	0.337 *	0.161	−0.069	1		
Exocanthion difference	0.176	0.179	0.096	0.064	1	
Commissure difference	0.056	0.316 *	0.193	−0.017	0.182	1

* Statistical test by Spearman correlation (* *p <* 0.05, ** *p <* 0.01).

## Data Availability

The data presented in this study are available on request from the corresponding author.
